# Respiratory sound energy and its distribution patterns following clinical improvement of congestive heart failure: a pilot study

**DOI:** 10.1186/1471-227X-10-1

**Published:** 2010-01-15

**Authors:** Zhen Wang, Brigitte M Baumann, Karen Slutsky, Karen N Gruber, Smith Jean

**Affiliations:** 1 Division of Critical Care Medicine, Robert Wood Johnson School of Medicine - University of Medicine and Dentistry of New Jersey - Cooper University Hospital, One Cooper Plaza, Camden, NJ 08103, USA; 2 Department of Emergency Medicine, Robert Wood Johnson School of Medicine - University of Medicine and Dentistry of New Jersey - Cooper University Hospital, One Cooper Plaza, Camden, NJ 08103, USA; 3 Department of Emergency Medicine, Beijing Shi-ji-tan Hospital, 10 Tie Yi Rd., Haidian District Beijing 100038, PR China

## Abstract

**Background:**

Although congestive heart failure (CHF) patients typically present with abnormal auscultatory findings on lung examination, respiratory sounds are not normally subjected to additional analysis. The aim of this pilot study was to examine respiratory sound patterns of CHF patients using acoustic-based imaging technology. Lung vibration energy was examined during acute exacerbation and after clinical improvement.

**Methods:**

Respiratory sounds throughout the respiratory cycle were captured using an acoustic-based imaging technique. Twenty-three consecutive CHF patients were imaged at the time of presentation to the emergency department and after clinical improvement. Digital images were created (a larger image represents more homogeneously distributed vibration energy of respiratory sound). Geographical area of the images and respiratory sound patterns were quantitatively analyzed. Data from the CHF patients were also compared to healthy volunteers.

**Results:**

The median (interquartile range) geographical areas of the vibration energy image of acute CHF patients without and with radiographically evident pulmonary edema were 66.9 (9.0) and 64.1(9.0) kilo-pixels, respectively (*p *< 0.05). After clinical improvement, the geographical area of the vibration energy image of CHF patients without and with radiographically evident pulmonary edema were increased by 18 ± 15% (*p *< 0.05) and 25 ± 16% (*p *< 0.05), respectively.

**Conclusions:**

With clinical improvement of acute CHF exacerbations, there was more homogenous distribution of lung vibration energy, as demonstrated by the increased geographical area of the vibration energy image.

## Background

Congestive heart failure (CHF) is one of the leading causes of frequent visits to the emergency department (ED) with a prevalence of 1-2% in the general population and a five year mortality rate after diagnosis reported at 60% in males and 45% in females [1]. Several methods are used to assess and monitor CHF patients during therapy including clinical symptoms, physical examination, echocardiography, brain natriuretic peptide (BNP) and chest radiography [2]. Since the advent of the stethoscope, clinicians have routinely listened to the sounds produced by a patient's internal organs, such as the heart and lungs, as a means of assessment and to diagnose pathology. Lung sounds (lung vibrations) are produced by airflow in and out of the lungs. In the past decade, there have been attempts to refine noninvasive acoustic data to better detect and monitor pulmonary abnormalities through the use of computerized lung sound analysis [3]. The theory behind using this type of analysis is that diseases affecting the lungs would result in alterations of lung vibration energy that may be too subtle to be detected on the skin surface using conventional methods. These altered vibrations may be due to changes in amount of vibration created due to increase or decrease in airflow, changes in the transmission of vibrations through the diseased lung parenchyma, or pleural space and heterogeneity of disease throughout the lung [3-7].

Computerized vibration imaging technology is able to record lung vibrations (energy) and convert the signals to a dynamic image of the lung in near real time. This technology has been studied recently for the detection of pleural effusion, and graft function in single lung transplant recipients [4,5]. To our knowledge, the relationship between vibration energy measured at the chest surface of the thorax of untreated and treated CHF has never been reported.

The aim of this pilot study is to document in detail the differences in respiratory sound patterns between normal individuals, CHF patients during acute exacerbations, and those same patients after clinical improvement.

## Methods

### Patients

The study protocol was approved by the Institutional Review Board, and informed consent was obtained from all participants. There were three groups of participants in this investigation. The first group consisted of acute CHF patients: Consecutive patients aged 18-85 years, who presented to the ED with acute shortness of breath and were diagnosed with CHF were eligible for inclusion in this study. All CHF patients were diagnosed by the attending emergency physician based on some combination of presenting complaint and symptoms, past medical history, physical examination findings, echocardiograph, BNP level and chest radiograph (Table 1).

**Table 1:** Subject characteristics.

Healthy volunteers and CHF patients groups	Healthy volunteers	Without pulmonary edema	With pulmonary edema	Pulmonary edema mechanically ventilated
N=	16	10	13	10

Age (mean ± SD)	49 ± 8.9	59.8 ± 19.4	59.5 ± 10.7	65.4 ± 11.9
Male	15 (94%)	8 (80%)	9 (69%)	3 (30%)
Female	1 (6%)	2 (20%)	4 (31%)	7 (70%)
Etiology				
Hypertension	0	2 (20%)	7 (54%)	3 (30%)
Ischemic heart disease	0	5 (50%)	6 (46%)	6 (60%)
Not specified	0	3 (30%)	0	1 (10%)
Chest x-ray findings				
Normal	16 (100%)	10 (100%)	0	0
Cardiomegaly	0	0	13 (100%)	10(100%)
Pulmonary edema	0	0	13 (100%)	10 (100%)
Pleural effusion	0	0	3 (23%)	8 (80%)
Echocardiograph				
Cardiomegaly	N/A	0	9 (69%)	10(100%)
LVEF < 40%	N/A	4 (40%)	10 (77%)	9(90%)
LV diastolic dysfunction	N/A	0	3 (23%)	2(20%)
Brain natriuretic peptide				
Normal (=< 100 ng/L)	0	0	1(8%)	0
Elevated (>100 ng/L)	0	10 (100%)	8(62%)	2(20%)
N/A	16 (100%)	0	4 (30%)	8 (80%)

Values are mean ± standard-deviation or n (%). LVEF, Left ventricular ejection fraction. LV, Left ventricular. N/A: Not available. SD: Standard deviation. Without pulmonary edema, without radiographically evident pulmonary edema. With pulmonary edema, with radiographically evident pulmonary edema. Pulmonary edema mechanically ventilated, Radiographically evident pulmonary edema in patients who were mechanically ventilated.

Results of chest radiograph and echocardiograph were based on official radiology and cardiology reports, respectively (see Table 1). The study patients were analyzed as two groups based on the presence or absence of radiographically evident pulmonary edema (REPE), (see Table 1). Patients with hemodynamic instability or coexisting pulmonary disease and those who were deemed unable to sit without assistance were unable to participate with the imaging procedure and therefore excluded.

The second group of study participants included mechanically ventilated CHF patients with the same ventilator settings and same tidal volumes before and after clinical improvement. This sample was enrolled to control for the variable tidal volumes. The third group was a control group of healthy subjects with no known cardiopulmonary disease and normal chest radiographs (as per official report).

### Recording Procedure and Data Analysis

For the CHF patients, respiratory sound data was acquired on the day of presentation to the ED and again on the day of discharge. For the mechanically ventilated CHF patients, respiratory sound data was acquired when chest radiographs showed pulmonary edema and again before extubation when radiographs demonstrated an improvement in pulmonary edema. All recordings were obtained with the subjects in the seated position. The tidal volumes of the non-ventilated patients in this study were not measured; patients were instructed to take comfortable deep breaths for all recordings. All measurements were performed by one individual who followed a standardized protocol to obtain vibrational images. This individual was also blinded to the clinical and radiologic information of subjects.

**Figure 1:**
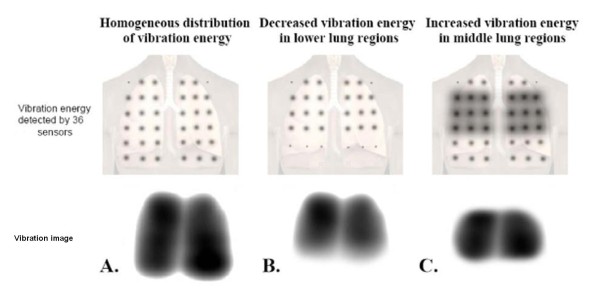
**Vibration energy image**. 36 vibration response imaging (VRI) sensors are spaced over the patient's back and detect vibrations during respiration. The size of the dots is a cartoon representation of the amount of vibration energy detected by that sensor. When the detected vibrations are uniform, the resulting VRI image will be large (A). When the detected vibration is less homogeneous, i.e. if the lower sensors have decreased vibrations (B) or if the middle sensors detect increased vibration (C), a smaller VRI image results. The visual geographical area is therefore determined not by intensity of vibration but by the distribution of intensity. VRI: vibration response imaging.

Respiratory sounds were captured using a vibration response imaging device (Deep Breeze™, Or-Akiva, Israel). This is a non-invasive computerized acoustic-based imaging technique that displays the geographic distribution of vibration energy of respiratory sounds throughout the respiratory cycle [4,5]. With this technique, 36 sensors (two arrays, one array over each lung) were adhered to the patient's back in a sitting position by a computer-controlled low vacuum and record the respiratory sound patterns. Subjects were instructed to take deep, comfortable breaths during 20 seconds of recording. Data collected by the sensors were processed and a grayscale video depicting the relative geographical distribution of respiratory sound was created. Each frame of the video was created from 0.17 seconds worth of data. The maximal energy frame was the frame in the video sequence that usually provided the most information on the distribution of lung vibration and usually approximated peak inspiration. The image from this frame was used for the area measurements. The image represents the relative distribution of vibration energy, not the absolute energy. A larger image indicates a more homogeneous distribution of vibration intensity throughout the lung and a smaller image a more focal distribution (Figure 1).

The area of the maximal energy frame image during inspiration was measured using the software *Image J*, a public domain Java image processing program inspired by National Institute of Health Image which traces the edge of the vibration energy image and objectively provides a digital measurement of the number of pixels in the traced image [8]. The automatic thresholding function (the same for all the images) used by *Image J *divides the image into visual signal (shade of grey to black) and background (white) and provides a numerical readout of area of the visual signal. In addition to image analysis of the maximal vibration energy frame, numerical maximal inspiratory vibration energy analysis was performed [4]. This analysis is not dependent on the image and allows total vibration energy to be compared before and after clinical improvement.

Respiratory sound generated vibration signals have very high signal intensity when compared to usual background noise and combined with the orientation of the sensor, allow pulmonary airflow to be the dominant signal in the gray-scale distribution. High energy artifacts from background noise due to patient movement against matrix framework or from sudden loud noises in the environment are occasionally encountered and easily identified in the image. These images were excluded from the analysis.

### Statistical Analysis

Wilcoxon signed rank test for paired and unpaired data (SPSS 11.5, SPSS Inc., Chicago, IL, USA) was used to analyze the data. Median and interquartile range (IQR) and mean ± standard deviation (SD) are reported. A pre-study power calculation was not possible due to unknown size of effect of pulmonary edema and treatment on amount and distribution of vibration energy. A *p *value less than 0.05 was considered statistically significant.

## Results

### Patients

A total of 23 consecutive CHF patients with shortness of breath at rest were enrolled in the study. Follow-up studies were obtained on the day of discharge following clinical improvement of CHF symptoms (no shortness of breath) and were not obtained for 4 patients due to timing of their discharge. In addition to the improvement of CHF symptoms such as shortness of breath, physical examination findings documented by the physicians were also used as objective measures of clinical improvement. All patients were discharged on the recommendation of the treating physician. The average hospital stay for CHF patients without and with REPE was 2.9 ± 1.6 days and 3.4 ± 1.4 days respectively (p > 0.05). CHF patients without and with REPE had a negative fluid balance during treatment of 1736 ± 945 ml/day and 1871 ± 763 ml/day, respectively (P > 0.05).

### Vibration energy images

**Figure 2:**
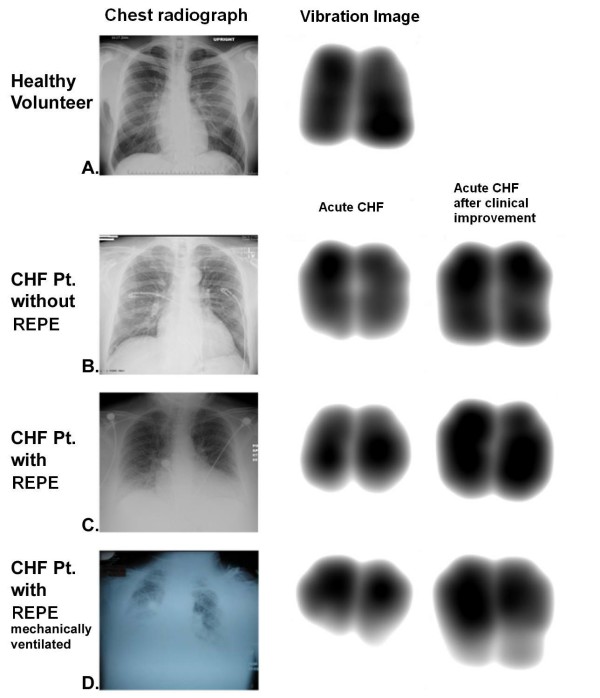
**Representative vibration response imaging images and chest radiographs**. A, Healthy volunteer. B, congestive heart failure (CHF) patients without radiographically evident pulmonary edema (REPE). C, CHF patients with REPE. D, Mechanically ventilated CHF patients with REPE. VRI, vibration response imaging. CHF, congestive heart failure. REPE, radiographically evident pulmonary edema.

Chest radiographs and vibration energy images for healthy volunteers and CHF patients on admission and after clinical improvement are shown in Figure 2. The images of healthy volunteers encompassed the entire imaging field and were symmetric (Figure 2A). For CHF patients without REPE, the vibration energy images were smaller (Figure 2B), and for CHF patients with REPE the images were even smaller. These effects were noted in both hemithoracies (Figure 2C).

### Geographic Area and Vibration Energy of Respiratory Sounds

**Figure 3:**
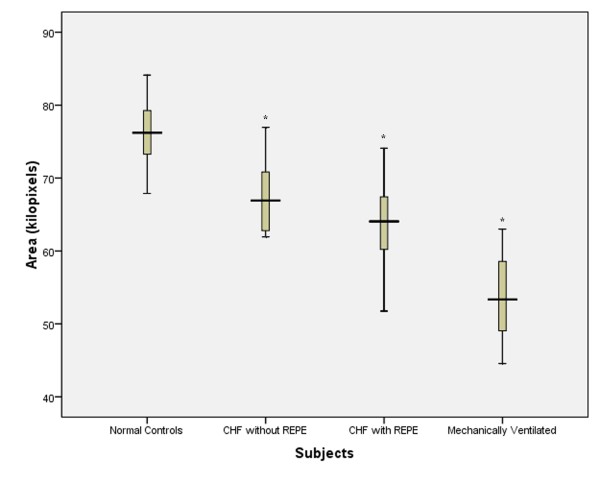
**Geographical area of vibration images during maximal inspiration in healthy volunteers, acute CHF exacerbation patients without and with REPE and CHF patients with REPE mechanically ventilated**. CHF, congestive heart failure. REPE, radiographically evident pulmonary edema. Boxes show median and interquartile ranges and I bars represent highest and lowest values.* = lower area values compared to healthy volunteers (P < 0.05).

**Figure 4:**
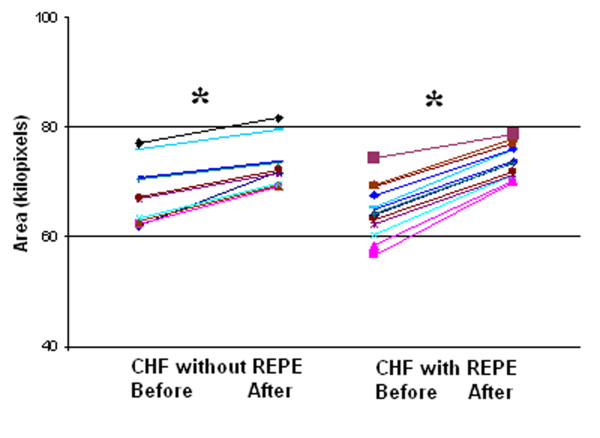
Geographical area of vibration images during maximal inspiration in acute CHF exacerbation patients without and with REPE on admission (Before) increased after clinical improvement (After) (*, P < 0.05). Each color/line represents a patient. CHF, congestive heart failure. REPE, radiographically evident pulmonary edema.

**Figure 5:**
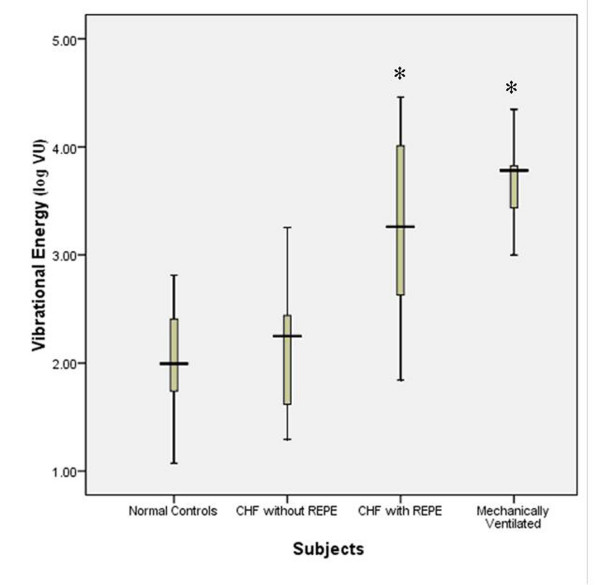
**Vibration energy during maximal inspiration in healthy volunteers, acute CHF exacerbation patients without and with REPE and CHF patients with REPE mechanically ventilated**. CHF, congestive heart failure. REPE, radiographically evident pulmonary edema.* = higher vibrational energy when compared to normal controls (P < 0.05).

**Figure 6:**
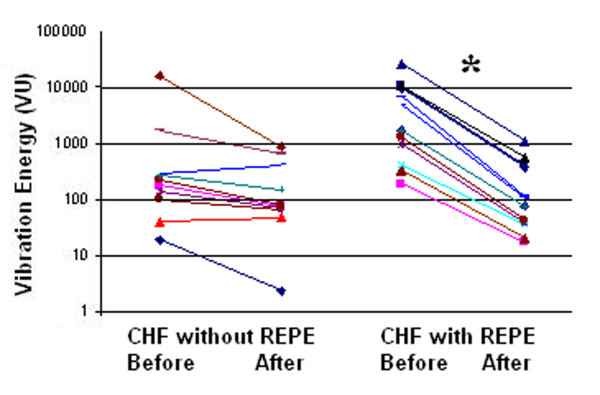
Vibration energy during maximal inspiration in acute CHF exacerbation patients without and with REPE on admission (Before) decreased after clinical improvement (After) (* = P < 0.05). Each color/line represents a patient. CHF, congestive heart failure. REPE, radiographically evident pulmonary edema.

The median geographic area of each maximal inspiratory vibration energy image was calculated. In healthy volunteers, themedian (IQR) geographical area of the vibration energy image was 76.2 (6.0) kilo-pixels. On admission, areas for CHF patients without REPE and those with REPE were66.9 (9.0) and 64.1(9.0), respectively (*p *< 0.05) (Figure 3). On admission, the geographical area in CHF patients without and with REPE was significantly lower compared to the geographical area of healthy volunteers (*p *< 0.05). After clinical improvement, the geographic area increased to 71.9 (12.0) and 73.4 (12.0) kilo-pixels in patients without REPE and with pulmonary edema, respectively (Figure 4). This corresponded to increases in area of 18 ± 15% (*p *< 0.01) and 25 ± 16% (*p *< 0.01), in the without REPE and with REPE patients, respectively. The total vibration energy values were calculated in each group on admission and were found to be significantly higher in CHF patients with REPE compared to those without REPE and healthy volunteers (Figure 5) (*p *< 0.05 between edema group and others). Total vibration energy decreased in CHF patients with REPE following clinical improvement by an average of 90 ± 11% (*p *< 0.01) but remained unchanged in CHF patients without REPE (Figure 6).

### Mechanically Ventilated Patients

**Figure 7:**
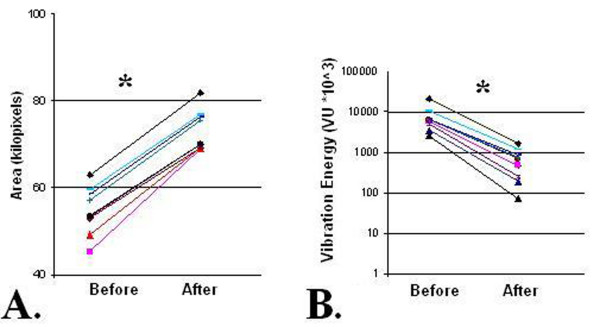
In mechanically ventilated CHF patients with radiographically evident pulmonary edema, geographical area (A) of vibration energy images increases and vibration energy (B) during maximal inspiration decreases after clinical improvement (* = P < 0.05). Each color/line represents a patient. CHF, congestive heart failure. REPE, radiographically evident pulmonary edema.

For mechanically ventilated CHF patients, all with REPE, the geographical area of the vibration energy images were smaller in size bilaterally and became larger after clinical improvement (Figure 2D) in a similar fashion as non-mechanically ventilated patients. The median geographical area was 52.5 ± 6.1 kilo-pixels on admission and increased to 73.9 ± 6 kilo-pixels with clinical improvement (Figure 5) (*p *< 0.01). This increase in size of the image was seen with tidal volume held constant. As in non-mechanically ventilated patients with REPE, total vibration energy also decreased following therapy (Figure 7) (*p *< 0.01).

## Discussion

In this initial exploratory study, we evaluated the visual display of respiratory sound patterns in patients with acute CHF exacerbation and after clinical improvement. Decreased geographical area of the vibration energy images were observed in CHF patients at presentation. With clinical improvement of CHF symptoms, the areas of these images increased. The decrease in geographic area was more pronounced in the presence of REPE. In CHF patients with REPE, total lung vibration energy decreased with clinical improvement.

The maldistribution of vibration energy of respiratory sounds in acute CHF exacerbations is likely produced by the distribution of pulmonary edema (which may or may not be radiographically evident). The vibration energy image represents the relative distribution of vibration energy, not the absolute energy. A larger image indicates a more homogeneous distribution of vibration intensity throughout the lung and a smaller image a more focal distribution. Because of how the vibration energy image is created, an increase in the size of the image after clinical improvement reflects a more homogeneous distribution of vibration intensity with decrease in lung water. The lack of homogeneity of lung vibration intensity throughout the chest in the presence of acute CHF might be explained by several mechanisms. Gravity-driven maldistribution of pulmonary edema may play a role [9-13] and heterogeneity of narrowed airways may also contribute to the heterogeneity of lung vibration images [14-20]. Due to the effects of gravity on lung water in patient who are in a seated position, it is expected that pulmonary edema would have more pronounced effects in the dependent lower lung regions causing vibration energy image to be smaller. This was the case in patients with pulmonary edema on erect chest radiographs (with or without mechanical ventilation).

The reason for the decreased area (distribution of vibration) seen peripherally in CHF patients is likely due to decreased transmission of breath sounds to peripheral lung tissue in the presence of pulmonary edema. This would in turn result in a smaller image due to decreased homogeneity of vibration intensity (less vibration peripherally and increased centrally) [4,5]. Lower airflow peripherally due to edema or narrowed airways could also result in a smaller image.

The increase in total vibration energy in patients with REPE is likely caused by more significant airflow obstruction resulting in narrowed airways and more turbulence. Although airflow obstruction in the setting of pulmonary edema has long been acknowledged by clinicians [12], the mechanisms responsible for this observation remain obscure. The elevation of pulmonary or bronchial vascular pressure likely results in reflex bronchoconstriction [13]. Other potential causes of airway narrowing include a geometric decrease in airway size from reduced lung volume, obstruction from intraluminal edema fluid, and bronchial mucosal swelling [13]. Some investigators [14,15], but not all [16], have found an increase in bronchial responsiveness to methacholine in patients with left ventricular dysfunction or mitral valve disease. The significance of this finding is not clear. Contrary to earlier reasoning, there is no evidence that engorged bronchovascular bundles directly compress small airways [17-19]. CHF associated airway edema may also cause larger airways to be narrowed producing increased turbulence and noise [12-18].

The edema may additionally cause smaller airways to close and reopen during inspiration; thereby, increasing noise [19,21-23]. Therapies that reduce interstitial pulmonary edema and pleural effusion result in reversal of the effects described above and would be expected to increase image area, as demonstrated in this investigation.

Another manifestation of CHF is cardiomegaly. Since the pulmonary system and the heart share one thoracic space, an enlarged heart would decrease the space available for the lungs to expand [24-26]. This may be an additional explanation for the decreased vibration energy areas. Cardiomegaly may cause decreased lung volume particularly in the lower lung fields.

In healthy volunteers, it has been shown that total vibration energy increases with increasing tidal volume [5]. Although the tidal volumes of the non-ventilated CHF patients in this study were not measured, the decrease in vibration energy seen with treatment is not likely due to decreased tidal volume because: 1) patients were instructed to take deep breaths for all recordings and larger volumes would be expected after clinical improvement [27] and 2) the increased work of breathing in CHF would likely cause respiratory muscle fatigue and shallow breathing (lower tidal volume) before therapy [28,29].

Nevertheless, to remove the confounding effects of tidal volume changes before and after clinical improvements, mechanically ventilated CHF patients were studied as a control group for same tidal volumes (not practical to quantitate tidal volume in the non-mechanically ventilated acutely dyspneic CHF patients), when REPE was present and again when the CHF had improved and REPE was reduced. In these patients, the geographical areas of the vibration energy images were also diminished, and increased with clinical improvements. The total vibration energy of respiratory sound was high and decreased following therapy. This group of patients had the same tidal volumes, ventilation modes and settings before and after clinically improvements. Therefore, these changes were not due to alterations in tidal volumes.

There were several limitations to this investigation. Because this was a preliminary study our sample size was small and we may not have been adequately powered for all of our analyses. We did not objectively measure pulmonary edema (e.g. via a pulmonary catheter). In a trial of critically ill patients, the use of a pulmonary artery catheter (PAC) was associated with increased mortality rates in patients with a lower severity of illness score while reducing mortality in the most severely ill [28]. A recently published analysis of 53,312 trauma patients revealed no beneficial effects of PAC monitoring on mortality in low-risk patients [30-32]. Since low-risk patients are unlikely to derive benefits from PAC monitoring, the use of such invasive monitoring would have placed participants in our investigation at increased and unjustifiable risk. As an alternative to invasive monitoring, we did note negative fluid balances for all CHF participants. Finally, inclusion of participants with pleural effusions may have led to confounding. Since patients with pulmonary effusion as well as pulmonary edema were pooled with patients who only had pulmonary edema, the possible confounding effects of effusion on the area and vibration energy is not known, although the CHF patients with and without pleural effusion showed similar trends. In most patients, a repeat chest radiograph was not deemed necessary by the physicians. A pre-study power calculation was not possible due to unknown size of effect of pulmonary edema and treatment on amount and distribution of vibration energy.

## Conclusions

In conclusion, with clinical improvement of acute exacerbation of CHF, there is a more homogenous distribution of vibration energy of respiratory sound. Respiratory sound analysis is non-invasive and also does not expose the patient to ionizing radiation. It can be conducted at the bedside and repeat measures are easily obtained. Based on our preliminary findings, this modality may be a useful adjunct to current methods in assessing improvement in acute CHF exacerbations.

## List of abbreviations

CHF: congestive heart failure; ED: emergency department; IQR: interquartile range; PAC: pulmonary artery catheter; REPE: radiographically evident pulmonary edema; SD: standard deviation; VRI: vibration response imaging.

## Competing interests

The authors declare that they have no competing interests.

## Authors' contributions

ZW and SJ carried out the VRI recordings. ZW, SJ and BB worked on the calculations of recordings and data analysis. ZW and SJ drafted the manuscript. BB, KS and KNG revised the manuscript critically for important intellectual content. All authors edited and approved the final manuscript.

## Pre-publication history

The pre-publication history for this paper can be accessed here:

http://www.biomedcentral.com/1471-227X/10/1/prepub
